# Clinical significance of combined detection of human papilloma virus infection and human telomerase RNA component gene amplification in patients with squamous cell carcinoma of the esophagus in northern China

**DOI:** 10.1186/2047-783X-18-11

**Published:** 2013-05-01

**Authors:** Yu-Feng Wang, Xin-Shuai Wang, She-Gan Gao, Qiang Chen, Yan-Tong Yang, Zhong-Yue Xiao, Xiu-Qing Peng, Xiu-Feng Hu, Qia-Yin Wang, Xiao-Shan Feng

**Affiliations:** 1 Department of Oncology, First Affiliated Hospital, Cancer Institute, Henan University of Science and Technology, Luoyang, Henan Province, 471003, China

**Keywords:** Squamous cell carcinoma of the esophagus, Prognosis, Human papilloma virus, Human telomerase RNA components gene

## Abstract

**Background:**

The aim of the study was to test for human papilloma virus (HPV) infection and human telomerase RNA component (hTERC) gene amplification in tissues derived from esophageal cancer, in esophagus displaying atypical hyperplasia and in normal tissue, and to analyze the relationship between them and discuss whether HPV infection and hTERC gene amplification play a role in the duration of survival of esophageal cancer patients.

**Methods:**

To test for HPV infection, surface plasma resonance was used after extracting and subjecting the DNA to PCR amplification. Measurement of hTERC gene amplification was performed by the fluorescence *in situ* hybridization technique.

**Results:**

The rates of HPV infection in the normal group, the atypical esophageal hyperplasia group and the cancer group were 0% (0/40), 10.00% (1/10) and 20.65% (19/92), respectively, with a statistically significant difference of *P* < 0.01. The hTERC gene amplification rate in normal tissue, grade I atypical hyperplastic tissue, grade II/III atypical hyperplastic tissue and esophageal cancer tissue were 0% (0/89), 15.38% (4/26), 47.06% (8/17) and 89.13% (82/92), respectively, with a statistically significant difference of *P* < 0.01. On follow-up of 92 patients, survival curves of the HPV-positive and HPV-negative groups were not significantly different (*P* > 0.05). Survival curves of the hTERC gene amplification-positive and hTERC gene amplification-negative groups were statistically significant (*P* < 0.05). A matching chi-square test showed that there was no correlation between HPV infection and hTERC gene amplification (*P* > 0.05).

**Conclusion:**

HPV infection may be one of many factors contributing to the development of esophageal cancer, but it does not influence prognosis. Amplification of the hTERC gene appears to influence certain features associated with postoperative survival in esophageal carcinoma patients.

## Background

China has the highest incidence and mortality of squamous cell carcinoma of the esophagus (ESCC) in the world. Patients with ESCC are most commonly diagnosed with locally advanced tumor stages. Early metastatic disease and late diagnosis are common reasons responsible for the poor clinical outcome of this tumor. The prognosis of ESCC is very poor because of the paucity of symptoms seen in these patients at the earliest stages of disease. Progressively increasing epidemiologic and biologic evidence indicates that human papilloma virus (HPV) infection is associated with the occurrence of ESCC [1-3]. In cervical cancer, the human telomerase RNA component (hTERC) gene may influence the integration of HPV into the cellular genome, accumulation of numerical chromosome aberrations and development of genomic instability with a consistent gain of chromosome arm 3q. Hopman and colleagues showed that both the genomic integration of oncogenic HPV and gain of the hTERC gene appeared to be important genetic events that were associated with the progression of uterine cervical dysplasia to an invasive cancer [4]. In ESCC, there is also a gain of chromosome arm 3q [5]. By taking into account that ESCC and cervical cancer are both squamous cell carcinomas, we explored whether HPV infection and the hTERC gene in ESCC display similar mechanisms in the pathogenesis of cervical cancer.

## Methods

### Materials

Ninety-two specimens of freshly frozen ESCC were collected from December 2007 through December 2008 at the First Affiliated Hospital of Henan University of Science and Technology. Esophageal cancer tissue, esophageal cancer side tissue (approximately 2 cm) and normal tissue were selected and made into sectioned wax pieces. Ninety-two cases of esophageal cancer tissue, 26 cases of grade I esophageal atypical hyperplastic tissue, 17 cases of grade II/III esophageal atypical hyperplastic tissue and 89 cases of esophageal normal tissue were selected, and their pathology was confirmed. Ten cases of esophageal atypical hyperplastic tissue and 40 cases of normal tissue were collected by endoscopic examination over the same period. This study was conducted in accordance with local ethics guidelines compliance with the Helsinki Declaration, the patients’ esophagus tissues used in this study were approved by the Medical Ethics Committee of the First Affiliated Hospital of Henan University of Science and Technology, Luoyang, China, reference no. 2010006.

### Methods and apparatus

DNA was isolated from frozen esophageal mucosa using the TIANamp Genomic DNA Kit (Tiangen Biotech (Beijing) Co., Ltd, Beijing, China). Excised tissue (20 μg) in liquid nitrogen was ground until the tissue was powder. Liquid nitrogen was allowed to evaporate and the powdered tissue added to buffer GA (200 μl), and then RNAase was added to a final concentration of 2 μg/μl, mixed and incubated for 30 minutes at 37°C. Proteinase K was added to a final concentration of 100 μg/μl and placed in a water bath for 3 hours at 56°C. Buffer GB (200 μl) was added, mixed and incubated for 10 minutes at 70°C. Ethanol was then added, and the solution mixed and transferred to a spin column that was closed and placed in the microcentrifuge. The mixture was centrifuged for 1 minute at 13,400 × *g*, the filtrate discarded and the spin column placed in the microcentrifuge. Buffer GD (500 μl) was added and again the mixture was centrifuged for 1 minute at 13,400 × *g*, the filtrate discarded and the spin column placed in the microcentrifuge. Buffer PW (600 μl) was added, and the mixture was centrifuged for 1 minute at 13,400 × *g*, the filtrate discarded and the spin column placed in the microcentrifuge. TE (50 μl) was then centrifuged for 2 minutes at 13,400 × *g*, and the filtrate collected. The DNA concentration was measured using a Nanodrop 1000 (Thermo Scientific, Waltham, MA, USA), an A_260_:A_280_ ratio of 1.75 to 2.05 indicating an absence of contaminating proteins. The isolated DNA was amplified by the HPV L1 consensus primers MY11 and MY09 [6].

The PCR processes were monitored through amplification of part of the human β-actin gene in replicate tubes with related primers [7]. Each PCR (50 μl) mixture consisted of 5 μl of 10× buffer (Mg^2+^), 2.5 mM MgCl_2_, 200 μM each dNTP, 0.2 μM my11/my09, 2.5 U Taq and 2 μl template DNA. Amplification without DNA template was used to monitor contamination in both the HPV and β-actin reactions. An HPV-positive sample was considered the positive control (Beijing GP Medical Technology Co., Ltd, Beijing China). The mixtures were incubated for 3 minutes at 94°C; then for 20 seconds at 94°C, 30 seconds at 55°C, 30 seconds at 72°C for 40 cycles; for 5 minutes at 72°C; and at 4°C on hold. A 10 μl aliquot of the PCR mixture was visualized by ethidium bromide staining after agarose gel electrophoresis. HPV-positive samples reveal a band of approximately 450 base pairs.

Surface plasma resonance (SPR) technology is a novel method to detect the HPV genotype. SPR exploits surface plasmons (special electromagnetic waves) that can be excited at certain metal interfaces, most notably silver and gold. When incident light is coupled with the metal interface at angles greater than the critical angle, the reflected light exhibits a sharp attenuation (SPR minimum) in reflectivity owing to the resonant transfer of energy from the incident light to the surface plasmon. The incident angle (or wavelength) at which the resonance occurs is highly dependent on the refractive index in the immediate vicinity of the metal surface. Binding of molecules at the surface changes the local refractive index, resulting in a shift of the SPR minimum angle. By monitoring changes in the SPR signal, it is possible to measure binding activities at the surface in real time.

The SPR instrument and the PCR and SPR kits were obtained from Beijing GP Medical Technology Co., Ltd. The peltier-based thermal cycler was obtained from the Hangzhou LongGene Scientific Instruments Co., Ltd (Hangzhou, Zhejiang, China). A probe panel based on the hTERC gene sequence was designed to evaluate the gain of chromosome 3q. The probes and the fluorescence *in situ* hybridization kit were obtained from Beijing GP Medical Technology Co., Ltd. Microscopic images were acquired using an Olympus B Microscope equipped with custom optical filters for 4,6-diamidino-2-phenylindole, spectrum green and spectrum red, as well as a Plan Apo objective. The fluorescence *in situ* hybridization IMSTAR 3.0 image analysis system was used (CYTOGEN, IMSTAR, Paris, France). All procedures were conducted in accordance with the product instruction manual.

### Evaluation criteria

The SPR gold chip was immobilized with 24 types of specific HPV probes targeting the L1 region of HPV genome. In addition, one positive control probe (specific to the human β-actin gene) and one negative control probe (unrelated to both HPV and human genome sequences) were also spotted on the gold chip. For an informative reaction, the positive control probe spot should yield a positive hybridization resonance unit, the negative control probe spot a negative hybridization resonance unit and the rest of the HPV probe spot different hybridization resonance units, depending on the HPV status existing in each specimen. A positive control signal value <40 or a negative control signal value ≥40 were indicative of obtaining an invalid result. A positive control signal value ≥40 and negative control signal value <40 were indicative of obtaining a valid result. For the 24 types of specific HPV probes, a probe signal ≥40 was considered HPV-positive and a signal <40 was considered HPV-negative. The cutoff value was set at 17.3 per 100 random nuclei displaying increased hTERC signals and/or tumor ploidy (for more detail see Results).

### Statistical analysis

For the evaluation of the data, SPSS 17.0 statistical software (SPSS Company, Chicago, IL, USA) was used to perform the chi-square test. The follow-up data obtained from different subgroups of patients with esophageal cancer were assessed by Kaplan–Meier survival analysis and use of log-rank hypothesis testing of the survival curves for the different groups. *P <* 0.05 was considered statistically significant.

## Results

**Figure 1:**
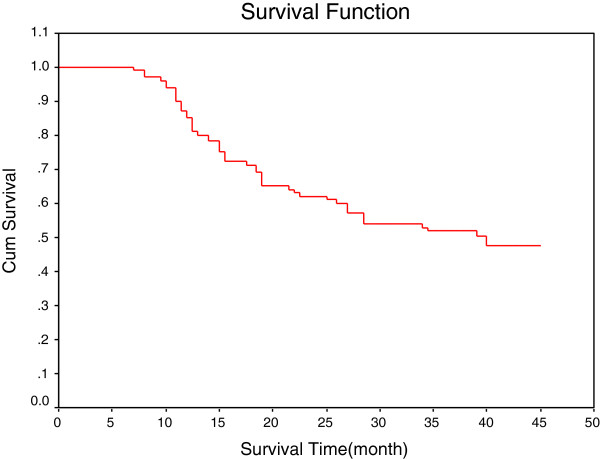
Survival curve of 92 patients.

**Figure 2:**
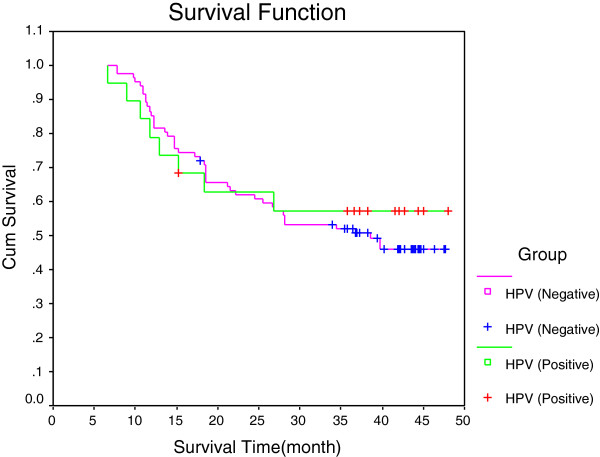
**Survival curves of human papilloma virus-negative and human papilloma virus-positive patients.** Human papilloma virus (HPV)-negative group, *n* = 73; HPV-positive group, *n* = 19.

Of 92 cases of cancer tissue, 10 cases of atypical hyperplasia tissue and 40 cases of normal esophageal mucosa, only 20 cases were found to test positive for HPV. The infection type included the high-risk types HPV18, HPV35, HPV51, HPV56, HPV66, HPV58, HPV68, HPV39, HPV81, HPV59 and HPV16, and the low-risk types HPV42 and HPV54. Mixed infection was found to exist in five specimens, and four specimens contained the HPV18 type. The main infection types were found to be HPV18, HPV35 and HPV51. The HPV infection rates of normal tissue, atypical hyperplasia of the esophagus and cancer tissue were found to be 0% (0/40), 10.00% (1/10) and 20.65% (19/92), respectively, with a statistically significant difference of *P* < 0.01. There was no significant correlation found between HPV infection and either gender, age, history of alcohol or tobacco use and family history (Table 1). Ninety-two patients were followed for 37 to 49 months after their operation, and the 3-year survival rate was found to be 51.94%. Survival curves are shown in Figure 1. The survival analysis showed that the 3-year survival rate in the HPV-positive group (19 patients) was 57.02%. The 3-year survival rate in the HPV-negative group (73 patients) was 50.70%. Comparison of both survival curves showed that they were not significantly different (*P* > 0.05). The survival curves for the HPV-negative group are shown in Figure 2.

**Table 1:** Relationship between human papilloma virus infection and clinical data

**Clinical data**	**Number of cases**	**Positive number**	**Percent positive**	***χ***^**2**^	***P *value**^**a**^
Gender					
Male	56	9	16.07	1.833	0.176
Female	36	10	27.78		
Age					
>60 years	47	12	25.53	1.396	0.237
≤60 years	45	7	15.56		
Smoke					
Yes	27	7	25.93	0.649	0.421
No	65	12	18.46		
Alcohol use					
Yes	32	6	18.75	0.108	0.742
No	60	13	21.67		
Family history					
Yes	19	5	26.32	0.469	0.494
No	73	14	19.18		

^a^*P* < 0.05 was considered statistically significant.

The signals were visually evaluated by screening the entire slide for the chromosome 3q-specific probe (using the spectrum red-specific optical filter). Cells with normal 3q signal numbers were recorded as diploid. Cells with abnormal 3q signal numbers were recorded in patterns in the relocation charts of the whole probe panel. Abnormal nuclei were defined by the recording of more than two hTERC signals and more than two centromeric region of chromosome 3 (CSP3) signals. This cutoff value was based on 35 normal tissues:

$$\text{Threshold}=\text{mean}\;\text{value}+\left(3\times \text{standard}\;\text{deviation}\right)$$

For normal tissue slides (*n* = 35), the numbers of hTERC gene amplification cells (>2 hTERC signals) were counted in 100 cells of random view, and the mean value for the 35 normal tissues was 7.1 (standard deviation 3.4). The cutoff value was set at 17.3. A case was considered hTERC gene amplification-positive for the 3q assay when >17.3% of the cells exhibited >2 hTERC signals.

**Figure 3:**
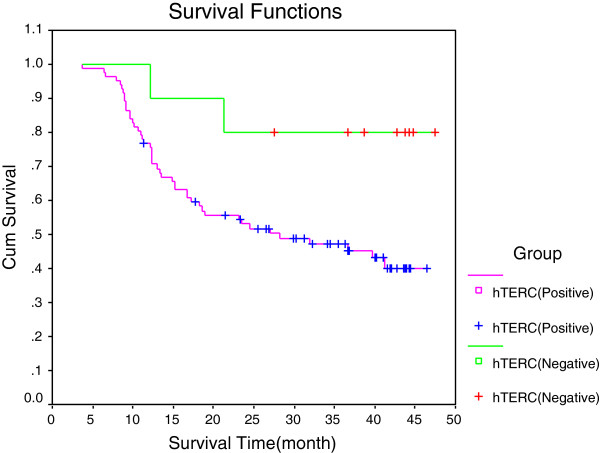
**Survival curves of human telomerase RNA component gene-negative and gene-positive patients.** Human telomerase RNA component (hTERC) gene-negative group, *n* = 10; hTERC gene-positive group, *n* = 82.

The hTERC gene amplification rates for the normal group, the group with grade I dysplasia, the group with grade II/III dysplasia and the esophageal cancer group were 0% (0/89), 15.38% (4/26), 47.06% (8/17) and 89.13% (82/92), respectively, and were statistically significantly different (*P* < 0.01). The gene amplification of hTERC in different differentiation grades, infiltration depth, pathology type and lymph node metastasis was found to not be significantly different (*P* > 0.05) (Table 2). Survival analysis showed that the hTERC gene-positive group (82 patients) displayed a 3-year survival rate of 47.29%. The hTERC gene-negative group (10 patients) displayed a 3-year survival rate of 80%. The survival curves for these groups were statistically significant (*P* < 0.05) (Figure 3).

**Table 2:** Relationship between clinical pathology and the human telomerase RNA component genes

**Clinical data**	**Number of cases**	**Positive number**	**Percent positive**	***χ***^**2**^	***P *value**^**a**^
Gross morphology				1.567	0.667
Medullar	47	43	91.49		
Fungating	28	24	85.71		
Ulcerative	13	12	92.31		
Constrictive	4	3	75.00		
Degree of histology				1.188	0.552
High	26	23	88.46		
Medium	49	45	91.84		
Low	17	14	82.35		
Invasion full-thickness				0.085	0.770
No	42	37	88.10		
Yes	50	45	90.00		
Lymph node metastasis				0.083	0.773
Yes	33	29	87.88		
No	59	53	89.83		

^a^*P* < 0.05 was considered statistically significant.

In 92 cases of esophageal carcinoma, 19 cases were found to be infected with HPV, and in the remaining 73 patients HPV infection could not be detected. In addition, hTERC gene amplification was detected in 82 cases, and in 10 cases hTERC gene amplification was not detected. There were also 15 cases in which not only the existence of HPV infection was confirmed but also hTERC gene amplification was detected. In 67 cases, the existence of hTERC gene amplification in the absence of HPV infection was found. By contrast, four cases were negative for hTERC gene amplification in the presence of HPV infection, and in six cases neither hTERC gene amplification nor HPV infection was found. The matching chi-square test showed that there was no correlation between HPV infection and amplification of hTERC genes (*P* > 0.05).

## Discussion

In 1982, Syrjanen and Pyrhonen first reported that HPV infection was associated with the occurrence of esophageal cancer [8]. Later, many groups studied the relationship between HPV infection and esophageal cancer. However, the conclusions drawn were inconsistent. Owing to differences in the detection methods used and the region of study, the incidence of HPV infection and the rate of esophageal cancer were reported to range from 8.70 to 78.11% [3,9]. By contrast, the infection rate of HPV in the normal population ranged from 0 to 59.76% [10], even in the same region. Additionally, the rates of HPV infection were very inconsistent between different researcher groups [11]. Shen and colleagues used HPV18E6/E7 to transfect human fetal esophageal epithelial cells and found that it could induce cellular immortalization [12]. They concluded that immortalized cells could arise following malignant transformation after several generations of cellular proliferation through mechanisms that depended on the synergistic effect of the cancer-promoting compounds.

In this study, we used SPR technology to detect HPV infection in samples from 92 esophageal cancer patients, 10 cases of esophageal atypical hyperplasia and 40 normal esophageal mucosa cases that were all obtained from Western Henan province. Infection with HPV included the high-risk types HPV18, HPV35, HPV51, HPV56, HPV66, HPV58, HPV68, HPV39, HPV81, HPV59 and HPV16, and the low-risk types HPV42 and HPV54. The main infection types were found to be HPV18, HPV35 and HPV51, and they existed as a mixed infection. Both HPV16 and HPV18 were closely associated with the identification of esophageal cancer in Linzhou city [13]. In this study, the results were not entirely consistent and indicated that different regions display different types of infection with HPV. There was also mixed infection found in esophageal cancer patients, which was similar to observations made in cervical cancer patients [14].

The infection rate of HPV in the esophagus of normal subjects, patients presenting with atypical hyperplasia and patients presenting with esophageal cancer were found to be 0%, 10.00% and 20.65%, respectively. It is possible that HPV plays a role in the occurrence of esophageal cancer. In the normal esophagus group, the infection rate was found to be 0%, which was concordant with prior observations in the literature for which the infection rate ranged from 0 to 59.76%. The survival curves of the positive and negative HPV infection groups were not significantly different (*P* > 0.05), suggesting that HPV infection may not be of prognostic utility in the evaluation of factors contributing to esophageal cancer.

The telomere–telomerase hypothesis for activation of telomerase is closely associated with cell immortalization and the occurrence or development of malignant tumors. The function of telomeres is to maintain chromosome stability and integrity. Telomerase comprises hTERC, human telomerase reverse transcriptase and human telomerase binding protein. Telomerase encodes the telomere repeat sequence that uses its own RNA as a template (hTERC). Historically, telomerase activity was thought to be closely associated with human telomerase reverse transcriptase. In recent years, hTERC has been of particular importance in regulating telomerase activity [15]. The hTERC gene, which encodes the telomerase RNA template, is located on chromosome 3q26.3, and the region of amplification was found to be 3q26 to 3q27. In our study, the rates of hTERC gene amplification in the normal group, the grade I atypical hyperplasia group, the grade II/III atypical hyperplasia group and the esophageal cancer group were found to be 0%, 15.38%, 47.06% and 89.13%, respectively, with a statistically significant difference of *P* < 0.01. These data indicate that the hTERC gene is involved in the carcinogenesis of esophageal epithelial cells and may play a role in the occurrence of esophageal cancer. Similar to observations with cervical atypical hyperplasia, hTERC gene amplification was closely associated with different grades of atypical esophageal hyperplasia [16,17].

The level of gene amplification of hTERC under conditions of different grades of differentiation, invasion depth, pathology type and lymph node metastasis was found to be statistically nonsignificant (*P* > 0.05). The hTERC gene may participate in the malignant transformation of esophageal epithelial cells, but it does not influence tumor invasion and metastasis. This finding might indicate that amplification of the hTERC gene is an early event in the development of esophageal cancer.

The survival curves for the hTERC gene amplification-positive and hTERC gene amplification-negative groups were statistically significant (*P* < 0.05). This observation suggested that hTERC gene amplification might be an independent risk factor for the prognosis of esophageal cancer. The hTERC gene is anticipated to become a new target to assist in the treatment of esophageal cancer.

There was no correlation between HPV infection and hTERC gene amplification in our study. Infection with HPV and the hTERC gene may have different pathogenic mechanisms in the development of esophageal and cervical cancer.

## Conclusion

HPV infection may be one of many factors contributing to the development of esophageal cancer, but it does not influence prognosis. Amplification of the hTERC gene appears to influence certain features associated with postoperative survival in esophageal carcinoma patients.

## Abbreviations

ESCC: Squamous cell carcinoma of the esophagus; HPV: Human papilloma virus; hTERC: Human telomerase RNA component; PCR: Polymerase chain reaction; SPR: Surface plasma resonance.

## Competing interests

The authors declare that they have no competing interests.

## Authors’ contributions

X-SF conceived and designed the experiments. Y-FW designed the experiments, prepared reagent and materials, performed the experiments, analyzed the data and wrote the paper. X-SW participated in analyzing the data and drafted the manuscript. S-GG analyzed the data. QC carried out the experiments. Y-TY carried out the experiments. Z-YX prepared reagent and materials. X-QP prepared reagent and materials. X-FH prepared reagent and materials. Q-YW prepared reagent and materials. All authors read and approved the final manuscript.

## References

[B1] Guo F Liu Y Wang X He Z Weiss NS Madeleine MM Liu F Tian X Song Y Pan Y Ning T Yang H Shi X Lu C Cai H Ke Y Human papillomavirus infection and esophageal squamous cell carcinoma: a case–control study Cancer Epidemiol Biomarkers Prev 2012 21 780 785 10.1158/1055-9965.EPI-11-1206 22337534

[B2] Gupta N Barwad A Rajwanshi A Kochhar R Prevalence of human papilloma virus in esophageal carcinomas: a polymerase chain reaction-based study Acta Cytol 2012 56 80 84 10.1159/000332909 22236750

[B3] Zhang QY Zhang DH Shen ZY Xu LY Li EM Au WW Infection and integration of human papillomavirus in esophageal carcinoma Int J Hyg Environ Health 2011 214 156 161 10.1016/j.ijheh.2010.11.001 21130683

[B4] Hopman AH Theelen W Hommelberg PP Kamps MA Herrington CS Morrison LE Speel EJ Smedts F Ramaekers FC Genomic integration of oncogenic HPV and gain of the human telomerase gene TERC at 3q26 are strongly associated events in the progression of uterine cervical dysplasia to invasive cancer J Pathol 2006 210 412 419 10.1002/path.2070 17054308

[B5] Hu N Clifford RJ Yang HH Wang C Goldstein AM Ding T Taylor PR Lee MP Genome wide analysis of DNA copy number neutral loss of heterozygosity (CNNLOH) and its relation to gene expression in esophageal squamous cell carcinoma BMC Genomics 2010 11 576 10.1186/1471-2164-11-576 20955586 PMC3091724

[B6] Hildesheim A Schiffman MH Gravitt PE Glass AG Greer CE Zhang T Scott DR Rush BB Lawler P Sherman ME Persistence of type-specific human papillomavirus infection among cytologically normal women J Infect Dis 1994 169 235 240 10.1093/infdis/169.2.235 8106758

[B7] Takenouchi N Yamano Y Usuku K Osame M Izumo S Usefulness of proviral load measurement for monitoring of disease activity in individual patients with human T-lymphotropic virus type 1-associated myelopathy/tropical spastic paraparesis J Neurol Virol 2003 9 29 35 10.1080/13550280390173418 12587066

[B8] Syrjanen KJ Pyrhonen S Demonstration of human papilloma virus antigen in the condylomatous lesions of the uterine cervix by immunoperoxidase technique Gynecol Obstet Invest 1982 14 90 96 10.1159/000299456 6288519

[B9] Gao GF Roth MJ Wei WQ Abnet CC Chen F Lu N Zhao FH Li XQ Wang GQ Taylor PR Pan QJ Chen W Dawsey SM Qiao YL No association between HPV infection and the neoplastic progression of esophageal squamous cell carcinoma: result from a cross-sectional study in a high-risk region of China Int J Cancer 2006 119 1354 1359 10.1002/ijc.21980 16615110

[B10] Yao PF Li GC Li J Xia HS Yang XL Huang HY Fu YG Wang RQ Wang XY Sha JW Evidence of human papilloma virus infection and its epidemiology in esophageal squamous cell carcinoma World J Gastroenterol 2006 12 1352 1355 16552800 10.3748/wjg.v12.i9.1352 PMC4124309

[B11] Cui MC Li Y He X Wang XL Wang LD Liu HT Study of human papillomavirus in biopsy tissue specimens of esophageal carcinomas in Linzhou city Zhonghua Shi Yan He Lin Chuang Bing Du Xue Za Zhi 2011 25 39 41 21789852

[B12] Shen ZY Cen S Xu LY Cai WJ Chen MH Shen J Zeng Y E6/E7 genes of human papilloma virus type 18 induced immortalization of human fetal esophageal epithelium Oncol Rep 2003 10 1431 1436 12883719

[B13] Wang X Tian X Liu F Zhao Y Sun M Chen D Lu C Wang Z Shi X Zhang Q Shen Z Li F Harris CC Cai H Ke Y Detection of HPV DNA in esophageal cancer specimens from different regions and ethnic groups: a descriptive study BMC Cancer 2010 10 19 10.1186/1471-2407-10-19 20078887 PMC2826296

[B14] Siegler E Lahat N Shiner M Mackuli L Aroudi Y Shapiro S Auslander R Lavie O [Human papillomavirus types in women with cervical cancer in Haifa District] Harefuah 2011 150 837 841 876 22428203

[B15] Min B Collins K An RPA-related sequence-specific DNA-binding subunit of telomerase holoenzyme is required for elongation processivity and telomere maintenance Mol Cell 2009 36 609 619 10.1016/j.molcel.2009.09.041 19941821 PMC2913470

[B16] Eid MM Nossair HM Ismael MT Amira G Hosney MM Abdul Rahman R Clinical significance of hTERC and C-Myc genes amplification in a group of Egyptian patients with cancer cervix Gulf J Oncol 2011 1 18 26 21724525

[B17] Zhang Y Wang X Ma L Wang Z Hu L Clinical significance of hTERC gene amplification detection by FISH in the screening of cervical lesions J Huazhong Univ Sci Technolog Med Sci 2009 29 368 371 10.1007/s11596-009-0321-z 19513624

